# Effects of Dietary Inclusion of Shrimp Paste on Growth Performance, Digestive Enzymes Activities, Antioxidant and Immunological Status and Intestinal Morphology of Hybrid Snakehead (*Channa maculata ♀* × *Channa argus ♂*)

**DOI:** 10.3389/fphys.2019.01027

**Published:** 2019-08-07

**Authors:** Haohang Fang, Jiajun Xie, Shiyu Liao, Tianyu Guo, Shiwei Xie, Yongjian Liu, Lixia Tian, Jin Niu

**Affiliations:** State Key Laboratory of Biocontrol, Institute of Aquatic Economic Animal and Guangdong Province Key Laboratory for Aquatic Economic Animals, School of Life Sciences, Sun Yat-sen University, Guangzhou, China

**Keywords:** shrimp paste, hybrid snakehead, growth performance, intestinal morphology, feed intake

## Abstract

A nutritional feeding experiment was conducted to evaluate the effects of shrimp paste on feeding attractiveness, growth performance, digestive enzyme activities, immune-related genes and intestinal morphology in hybrid snakehead (*Channa maculata ♀* × *Channa argus ♂*). Two diets were formulated with or without shrimp paste supplementation (D1:0% and D2: 3%) to feed fish for 8 weeks. Results showed that growth performance (FBW, WG and SGR) and feed intake (FI) significantly increased with shrimp paste supplemented (*P* < 0.05), while FCR and SR of hybrid snakehead fed diets supplemented with shrimp paste or not showed no significant difference (*P* > 0.05). Gut lipase and amylase activities were significantly higher in diet supplemented with shrimp paste than that in control one (*P* < 0.05). Hepatic antioxidant statuses of hybrid snakehead fed dietary shrimp paste or not showed no significant differences in total antioxidant capacity, malondialdehyde and superoxide dismutase of fish (*P* > 0.05). Results showed that fish fed diet with shrimp paste supplemented did not show significant difference in expression of GR, IκB, P65 and IL8 than that in control group (*P* > 0.05). There are significantly more goblet cells in shrimp paste supplemented diet than that in control diet (*P* < 0.05). However, villi length and muscle thickness showed no significant difference compared to control diet (*P* > 0.05). The results indicated that dietary 3% shrimp paste supplementation improved the growth performance of hybrid snakehead by enhancing feed intake (FI) while made no difference to antioxidant capacity and immunity.

## Introduction

Carnivorous fish usually require well above 30% protein for optimum growth (Lindner et al., 1995) and fishmeal is the main protein source for carnivorous fish in commercial feed. As a primary high-quality protein source, fishmeal consists of more than 60% crude protein and full of vitamins, minerals and other nutrients (Riche, 2015). One of the reasons that fishmeal become the main source of protein ingredient in aquatic feed is because of its palatability for aquatic animal (Alexis and Nengas, 2001). However, the use of fishmeal is limited in aquafeed production because of the high demand and lack of fish stocks, which constrain the continued development of aquaculture (Tacon et al., 2011). Since it is unlikely to produce fishmeal by a large margin beyond the current need, aquaculture production may depend on inclusion of alternative protein sources, like plant protein sources (El-Haroun et al., 2012). Nevertheless, the use of the plant materials is mainly restricted by the presence of anti-nutritional ingredients and lower protein quality (Zhang et al., 2012). Besides, high content of plant protein sources can result in lower feed intake (FI) caused by low feed palatability (Nunes et al., 2006). To solve these problems, attractants was mainly used to enhance the utilization of feed (Tusche et al., 2011). Currently, there were lots of studies about the feed attractants published for aquatic animals, such as krill meals, fish and krill hydrolysates, squid meal, betaines, amino acids, AMP, or other animal based meals (Coman et al., 1996; Nunes et al., 2006; Derby et al., 2016). However, use of shrimp paste as a feed attractant is rarely reported in aquatic animals. Shrimp paste, as one of feed attractants, might be significant dietary sources of long chain n-3 polyunsaturated fatty acids, rich in free amino acids, nucleotides, amines and nucleosides (Montaño et al., 2001) and has strong shrimp odor (Adnan, 1984).

Snakeheads has been one of the most commercially important fish species for aquaculture in China for the fast growth, delicious taste, tolerance to inferior water quality and resistance to diseases (Hossain et al., 2008). The main farmed snakehead species in China include northern snakehead (Channa argus), blotched snakehead (*Channa maculata*) and hybrid snakehead (*Channa maculates* × *Channa argus*) (Sagada et al., 2017). Northern snakehead (*C. argus*) is native to the Yangtze River (Zhou et al., 2012), while blotched snakeheads (*C. maculata*) is native to Pacific coastal drainages in northern Vietnam and southern China, mainly located in Guangdong Province (Gong et al., 2014). Recently, the hybrid snakehead has gained popularity because of its rapid growth compared to those of *C. argus* and *C. maculate* (Shu-Ren et al., 2013). As a carnivorous fish, hybrid snakehead requires high content of protein in feed, being fishmeal usually considered as the most adequate protein source (Zhang et al., 2017). Currently, researches on better use of protein supplementing with feed attractant on hybrid snakehead are quiet fewer. Therefore, the current study was conducted to evaluate the effect of dietary shrimp paste in practical diets on the feed attractiveness, growth performance and digestive enzyme activities, antioxidant and immunological status and intestinal morphology of hybrid snakehead (*C. maculata* × *C. argus*).

## Materials and Methods

### Diet Preparation and Dietary Treatments

In this study, two isonitrogenous and isoenergetic practical diets were formulated supplementing with or without shrimp paste (D1: 0%; D2: 3%) (Table 1). The proximate composition of shrimp paste was shown in Table 2. The method of diet preparation was the same as described by Niu et al. (2014). The diets were air dried and stored −20°C until fed.

**TABLE 1:** Ingredients and proximate composition of the two experimental diets (%).

	**D1**	**D2**
Fish meal^1^	25	25
Soy protein concentrate^2^	6	6
Wheat flour^3^	17.46	20.47
Dehulled soybean meal^4^	22	22
Chicken powder^5^	6	6
Brewer yeast^6^	5	5
Peanut bran^7^	6	6
Soya lecithin^8^	1.5	1.5
Soya oil^9^	1	1
Fish oil^10^	2	2
Choline (50Ca(H_2_PO_4_)_2_^12^	2	2
Vitamin C^13^ 1	0.1	0.1
Vitamin premix^14^	1	1
Mineral premix^15^	1	1
DL-Met^16^	0.05	0.05
Lys-HCL (78%)^17^	0.2	0.2
Threonine^18^	0.19	0.18
Shrimp paste^19^	0	3
Sum	100	100
**Nutrient levels**		
Moisture	10.28	10.51
Crude protein	40.78	41.13
Crude lipid	6.83	7.95
Ash	11.27	11.73

*1: Imported from Australia. Protein: 68.74%; lipid: 7.56%. 2: Guangzhou Chengyi Company Ltd., Guangzhou, China. Protein: 65.3%; lipid: 3.24%. 3: Guangzhou Chengyi Company Ltd., Guangzhou, China. Protein: 12.49%; lipid: 2.8%. 4: Guangzhou Chengyi Company Ltd., Guangzhou, China. Protein: 47.86%; lipid: 1.7%. 5: Guangzhou Chengyi Company Ltd., Guangzhou, China. Protein: 63%; lipid: 10%. 6: Guangzhou Chengyi Company Ltd., Guangzhou, China. Protein: 46%; lipid: 1%. 7: Guangzhou Chengyi Company Ltd., Guangzhou, China. Protein: 50%; lipid: 7%. 8-18: Guangzhou Chengyi Company Ltd., Guangzhou, China. 14: Vitamin premix (mg or g/kg diet) thiamin 25 mg; riboflavin, 45 mg; pyridoxine HCl, 20 mg; vitamin B12, 0.1 mg; vitamin K3, 10 mg; inositol, 800 mg; pantothenic acid, 60mg; niacin acid, 200mg; folic acid, 20 mg; biotin, 1.20 mg; retinol acetate, 32 mg; cholecalciferol, 5mg; alpha-tocopherol, 120 mg; ascorbic acid, 2000mg; choline chloride, 2500 mg; ethoxyquin 150 mg, wheat middling, 18.52 g (Zhang et al., 2017). 15: Mineral premix (mg or g/kg diet): NaF, 2 mg; KI, 0.8 mg; CoCl2⋅6H2O (1%), 50 mg; CuSO4⋅5H2O, 10 mg; FeSO4⋅H2O, 80 mg; ZnSO4⋅H2O, 50mg; MnSO4⋅H2O, 60 mg; MgSO4⋅7H2O, 1200 mg; Ca(H2PO3)2⋅H2O, 3000 mg; NaCl, 100 mg; Zoelite, 15.45 g (Zhang et al., 2017). 19. The shrimp paste is offered by Shunde Xinfuda Bioengineering Company Ltd., Shunde, China. The ingredients and proximate analysis are presented on a dry matter basis.*

**TABLE 2:** Proximate composition of the shrimp pasted used in the present study.

**Composition**	**Content**
Moisture	40%
Crude protein	32–35%
Crude lipid	8%
Ash	6–15%
Animo acid	30%
Cholesterol	56 mg/kg
EAA/TAA	40.5%
EAA/NAA	68.08%
Glu	6.14%
Asp	3.77%
Gly	2.57%

*The shrimp paste is offered by Shunde Xinfuda Bioengineering Company Ltd., Shunde, China. Briefely, shrimp shells were removed and the carcases were enzymatic hydrolyzed. The zymolyte were separated, concentrated and sterilized to be the used shrimp paste. EAA: essential amino acid; TAA, total amino acid; NAA, non-essential amino acid; Glu, glutamic acid; Asp, aspartic acid; Gly, glycine.*

### Animal Rearing and Experimental Procedures

The feeding trial was conducted at an experimental station of Sun Yat-sen University (Guangzhou, Guangdong). Prior to the start of the trial, (*C. maculata* × *C. argus*) were acclimated to a commercial diet for 2 weeks and were fed twice daily to apparent satiation. At the beginning of the feeding trial, the fish were starved for 24 h, weighed after being anesthetized with 10 mg L-1 eugenol (Shanghai Medical Instruments Co., Ltd., Shanghai, China), and then fish with similar size (initial body weight 73.16 ± 0.40 g) were randomly allotted to 6 tanks (170L; three cages per diet treatment); each tank was stocked with 20 fish. Each experimental diet was randomly assigned to three tanks. The feeding frequency was twice daily at 8:00 and 16:00 and lasted for 8 weeks.

### Sample Collection

At the end of the feeding trial, fish were starved for 24 h, anesthetized with 10 mg L-1 eugenol (Shanghai Medical Instruments Co., Ltd., Shanghai, China) and then weighed and counted the total number. Eight fish from each tank were randomly collected. Two fish were collected for measuring the whole body composition. Six fish were used to obtain weights of liver, viscera and whole body for the biometric parameters. Livers and foreguts were rapidly removed and frozen in the liquid nitrogen for analysis of enzymes and gene expression. Foreguts were collected in Bouin’s solution for paraffin sectioning.

### Biochemical Analysis

Feed and whole fish were frozen dried and then grounded. Moisture, crude lipid, crude protein and crude ash of the feed and fish were determined using standard methods (Kavanagh, 2010).

### Antioxidant Capacity Analysis and Digestive Enzymes Analysis

Hepatic and intestinal samples were homogenized in ice-cold phosphate buffer (1:10 dilution) (phosphate buffer; 0.064 M, pH 6.4). The homogenate was then centrifuged for 15 min (4°C, 1200 g), and aliquots of the supernatant were used to quantify antioxidant status and digestive enzymes analysis. All indices were measured with commercial assay kits (T-AOC, A015-1; SOD A001-1-2, MDA, A003-1-2, Lipase, A054-2-1; Amylase, C016-2-1) (Nanjing Jiancheng Bioengineering Institute, Nanjing, China) in accordance with the instructions of the manufacturer.

### Quantitative Real Time PCR Analysis

Total RNA was extracted from liver using Trizol^®^ reagent (Invitrogen, United States). The cDNA was synthesized using a PrimeScript^TM^ RT reagent kit with gDNA Eraser (Takara, Japan), according to the manufacturer’s instructions. Real-time PCR for the target genes were performed using a SYBR^®^ Premix Ex Taq^TM^ II (Takara, Japan) and quantified on the LightCycler 480 (Roche Applied Science, Basel, Switzerland). The primers were showed in Table 3.

**TABLE 3:** Sequences of primers used in this study.

**Primers**	**forward/reverse (5′to 3′)**
GR-F	GGGAAAGACCAGGACTCATA
GR-R	TTCTTGGTTTTCCGTGCTTC
HSP70-F	ATTTTGAATGTGTCTGCGGT
HSP70-R	ACTTGCTGATGATGGGGTTA
IL-8-F	GAGTCTGAGCAGCCTGGGAGT
IL-8-R	CTGTTCGCCGGTTTTCAGTG
NF-κB p65-F	CAGCCAAAACCAAGAGGGAT
NF-κB p65-R	TCGGCTTCGTAGTAGCCATG
IκBα-F	AAAATGTTACCGTGCCAGGAC
IκBα-R	ATGTATCACCGTCGTCAGTC
β-actin-F	CACTGTGCCCATCTACGAG
β-actin-R	CCATCTCCTGCTCGAAGTC

*GR, glucocorticoid receptor; HSP70, heat shock protein 70; IL-8; interleukin-8; NF-κB p65, NF-κB p65 subunit; IκBα, inhibitor of NF-κBα.*

### Intestinal Morphology

Samples fixed in Bouin solution were dehydrated in ethanol, equilibrated in xylene and embedded in paraffin according to the method described by Krogdahl et al. (2003). The paraffin blocks was sectioned (5 μm) in serial sagittal section using a Leica RM 2135 rotary microtome and stained with hematoxylin and eosin (H and E). The sections were examined using a light microscope with villi length and muscle thickness measured. Photographs were taken with an Olympus digital camera attached to the microscope. 10 random villi from each segment were measured.

### Calculations and Statistical Analysis

The following variables were calculated:

Weight gain rate (WG, %) = 100 × (final body weight-initial body weight)/initial body weight;

Specific growth rate (SGR,% day-1) = 100 × (Ln final individual weight-Ln initial individual weight)/number of days;

Feed conversion ratio (FCR) = dry diet fed/wet weight gain;

Survival rate (%) = 100 × (final number of fish)/(initial number of fish);

Viscerosomatic index (VSI,%) = 100 × (viscera weight, g)/ (whole bodyweight, g);

Hepatosomatic index (HSI,%) = 100 × (liver weight, g)/(whole body weight, g);

Condition factor (CF, g/cm3) = 100 × (body weight, g)/(body length, cm3);

All data are presented as means ± S.E.M. and subjected to independent-sample *t*-test to test the effects of experimental diets using the software of the SPSS for windows (ver 16.0, U.A.S). Statistical significance was examined at *P* < 0.05 unless otherwise noted.

## Results

### Growth Performance, Feed Utilization, Survival Rate and Biometric Parameters

Growth performance, feed utilization and biometric parameters of hybrid snakehead fed dietary shrimp paste are shown in Table 4. Results showed that growth performance (FBW, WG, and SGR) significantly increased with shrimp paste supplemented (*P* < 0.05). Survival rate showed the same trend as the growth performance but without significant difference (*P* > 0.05). Feed conversion ratio (FCR) of snakehead fed diet supplemented with shrimp paste showed no significant difference with that in control group (*P* > 0.05), while FI of snakehead fed diet supplemented with shrimp paste was significantly higher than that in control group (*P* < 0.05). There was no significant difference among hepatosomatic indices (HSI), visceral somatic indices (VSI) and condition factor (CF) between the two different diet treatments.

**TABLE 4:** Growth performance and biometric parameters of hybrid snakehead (*Channa maculata* × *Channa argus)* fed diets with or without supplementation of shrimp paste.

	**D1**	**D2**
IBW/g	72.7 ± 0.59	73.6 ± 1.23
FBW/g	98.7 ± 4.98a	122.5 ± 8.68b
SGR/%⋅d-1	0.54 ± 0.10a	0.91 ± 0.11b
WG/%	35.8 ± 7.96a	66.5 ± 10.9b
SR/%	93.3 ± 5.77	98.3 ± 2.89
FI(g/100gBW/d)	1.01 ± 0.98a	1.38 ± 0.26b
FCR	1.77 ± 0.07	1.56 ± 0.13
VSI/%	4.80 ± 0.74	4.42 ± 1.19
HSI/%	1.45 ± 0.35	1.19 ± 0.43
CF/%	1.34 ± 0.07	1.29 ± 0.11

*Values are mean ± SEM of three replicates, and values in the same row with different letters are significantly different (*P* < 0.05). Initial body weight (IBW,%) = 100 × initial body weight/initial number of fish. Final body weight (FBW,%) = 100 × final body weight/final number of fish. Weight gain rate (WG,%) = 100 × (final body weight-initial body weight)/initial body weight. Specific growth rate (SGR,% day-1) = 100 × (Ln final individual weight-Ln initial individual weight)/number of days. Feed conversion ratio (FCR) = dry diet fed/wet weight gain. Survival rate (%) = 100 × (final number of fish)/(initial number of fish). Viscerosomatic index (VSI, %) = 100 × (viscera weight, g)/(whole bodyweight, g). Hepatosomatic index (HSI, %) = 100 × (liver weight, g)/(whole body weight, g). Condition factor (CF, g/cm3) = 100 × (body weight, g)/(body length, cm3).*

### Whole Body Composition

Whole body composition of hybrid snakehead fed dietary shrimp paste is shown in Table 5. There were no significant difference in whole body composition of fish between the two diet treatments (*P* > 0.05).

**TABLE 5:** Whole-body compositions (% dry weight) of hybrid snakehead (*Channa maculata* × *Channa argus)* fed diets with or without supplementation of shrimp paste.

	**D1**	**D2**
Moisture	73.34 ± 0.45	71.93 ± 1.15
Crude lipid	25.16 ± 1.94	24.53 ± 0.80
Crude protein	61.38 ± 1.16	61.96 ± 0.68
Ash	16.04 ± 0.27	15.62 ± 0.37

*Values are mean ± SEM of three replicates, and values in the same row with different letters are significantly different (*P* < 0.05).*

### Hepatic Antioxidant Status Analysis

Hepatic antioxidant statuses of hybrid snakehead fed dietary shrimp paste or not are shown in Table 6. There were no significant difference in total antioxidant capacity (T-AOC), malondialdehyde (MDA) and superoxide dismutase (SOD) of fish between the two diet treatments (*P* > 0.05).

**TABLE 6:** Hepatic antioxidant statuses of hybrid snakehead (*Channa maculata* × *Channa argus)* fed diets with or without supplementation of shrimp paste.

	**D1**	**D2**
T-AOC (U/mg protein)	0.21 ± 0.03	0.24 ± 0.02
SOD (U/mgprot)	95.83 ± 8.29	91.08 ± 11.24
MDA (nmol/ml)	0.23 ± 0.05	0.28 ± 0.02

*Values are mean ± SEM of three replicates, and values in the same row with different letters are significantly different (*P* < 0.05). T-AOC, total antioxidant capacity; SOD, superoxide dismutase; MDA, malondialdehyde.*

### Gut Digestive Enzymes Analysis

Gut digestive enzymes analysis of hybrid snakehead fed dietary shrimp paste or not are shown in Table 7. Gut lipase and amylase activity were significantly higher in diet supplemented with shrimp paste than that in control group (*P* < 0.05).

**TABLE 7:** Intestinal digestive enzyme activity of hybrid snakehead (*Channa maculata* × *Channa argus)* fed diets with or without supplementation of shrimp paste.

	**D1**	**D2**
Amylase/U^*^mgprot-1	0.47 ± 0.07a	0.81 ± 0.05b
Lipase/U^*^mgprot-1	33.47 ± 8.11a	57.01 ± 5.95b

*Values are mean ± SEM of three replicates, and values in the same row with different letters are significantly different (*P* < 0.05).*

### Intestinal Genes Expression Level

The relative genes expression level of hybrid snakehead fed diets with or without supplementation of shrimp paste are showed in Table 8. Results showed that there were no significant difference in GR (glucocorticoid receptor), HSP70, IL8, IκB and P65 gene expression level between the two diets (*P* > 0.05).

**TABLE 8:** The relative genes expression level of hybrid snakehead (*Channa maculata* × *Channa argus)* fed diets with or without supplementation of shrimp paste.

	**D1**	**D2**
GR	1.05 ± 0.26	0.70 ± 0.04
HSP70	1.00 ± 0.07	0.62 ± 0.20
IL8	1.02 ± 0.25	0.83 ± 0.03
IκB	1.02 ± 0.16	0.79 ± 0.05
P65	1.05 ± 0.14	0.78 ± 0.04

*Values are mean ± SEM of three replicates, and values in the same row with different letters are significantly different (*P* < 0.05). GR, glucocorticoid receptor; HSP70, heat shock protein 70; IL-8, interleukin-8; NF-κB p65, NF-κB p65 subunit; IκBα, inhibitor of NF-κBα.*

### Intestinal Morphology

**FIGURE 1:**
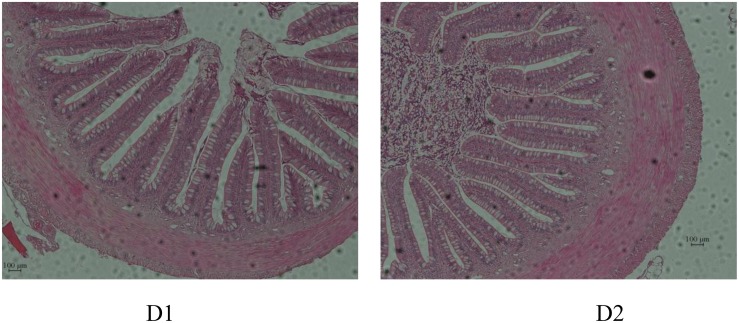
Comparison of gut morphology in hybrid snakehead (*Channa maculata* × *Channa argus)* fed diets with and without supplementation of shrimp paste.

Intestinal morphology is presented in Table 9 and Figure 1. Results showed that gut morphology was slightly changed by dietary shrimp paste. With shrimp paste supplemented in diet, there were significantly more goblet cells than that in control diet (*P* < 0.05). However, the villi length and muscle thickness showed no significant difference between the two groups (*P* > 0.05).

**TABLE 9:** Gut morphology of hybrid snakehead (*Channa maculata* × *Channa argus)* fed diets with or without supplementation of shrimp paste.

	**D1**	**D2**
Muscle thickness	198.33 ± 46.39	217.72 ± 39.80
Villi length	405.70 ± 69.56	399.78 ± 56.30
Goblet cells	447.00 ± 9.44a	529.00 ± 22.82b

*Values are mean ± SEM of three replicates, and values in the same row with different letters are significantly different (*P* < 0.05).*

## Discussion

The major components of feed attractants are shown to be water-soluble and relatively small, such as amino acids, mainly alanine, taurine, arginine, glutamic acid, glycine and alanine; small peptides, nucleotides and nucleosides, amines and quaternary ammonium bases, for example betaine (Lee and Meyers, 2010). Research showed that once the ingredients was soluble in water and had a high level of small peptides, especially high proportion of nucleotides and amino acids, the palatability and attractability aspects of the protein source to aquatic animals is better (Suresh et al., 2011). Aquatic animals normally rely on chemosensory systems to identify water-soluble chemicals, locate food and then ingest it (Derby and Sorensen, 2008). The chemical compounds in shrimp paste such as nucleotides and free amino acids are recognizable to the chemosensory systems for fish to locate and ingest food (Suresh et al., 2011). There were previous studies showing that marine animal additives could improve the performance of feed pellets in aquaculture. Williams et al. (2005) showed that krill and shrimp head meal supplemented in a basal diet improved the *Penaeus monodon* growth performance in a dose-dependent manner. Sanchez et al. (2005) showed that krill meal improved attractability of a feed and thus enhance the palatability of the diet for pacific white shrimp. These studies showed that marine animal additives acting as the feed attractants can enhance feed performance via the way of improving the attractability and palatability by stimulating appetitive behavior, for example, arousing, search initiating and locating the food and therefore enhancing feed consumption (Derby et al., 2016). Besides, enterocytes are more likely to digest and absorb the protein hydrolysates which consist of amino acids and low molecular-weight peptides compared to the high-molecular-weight macromolecules (Önal and Langdon, 2009). The low molecular-weight peptides are known to have excellent texture and viscosity and are highly digestible, which facilitate the uptake of nutrients (Bhaskar et al., 2007; Ospina-Salazar et al., 2016). The present results indicated that hybrid snakehead fed the shrimp paste diet had the higher growth performance than the control treatment, mainly by increasing the FI, which can be speculated that shrimp paste acted as the feed attractants. Besides, the better growth performance may also be related to the ability of shrimp paste regulating the production of enzyme activities in fish and thus exerting the effect on the digestion. The ability of aquatic animals to utilize the ingested nutrients mainly relies on the presence of digestive enzymes, which is also considered to be indicators of the fish absorptive and digestive capacity (Deguara and Jauncey, 2010). Lipases are the enzymes which catalyze the hydrolysis of ester bonds in substrates, such as triacylglycerol, thereby releasing free fatty acid and glycerol and providing energy (Wong and Schotz, 2002). In the present study, fish fed diets with shrimp paste supplemented had higher lipase and amylase activities, suggesting that shrimp paste supplemented diets up-regulated the lipid and carbohydrate metabolism in the present study. Increasing activities of digestive enzymes may have effects on the improved growth performance of fish in shrimp paste supplemented treatment.

Except for digestive enzyme activities, the morphology and structure of the intestine are crucial for nutrient absorption and the maintenance of normal intestinal functions (Gao et al., 2013; Vizcaíno et al., 2014). The villi length in a way reflects the function of the intestinal wall (Emami et al., 2012), led to better nutrient absorption and better growth performance (Al-Fataftah and Abdelqader, 2014). Muscle thickness as well plays a role in intestinal digestion and absorption. Increased muscle thickness may enhance intestinal digestion and absorption ability (Chen and Wang, 2013). However, in the present study, fish fed diets with shrimp paste supplemented made no difference to villi length and muscle thickness, indicating that shrimp paste did not improve the morphology and structure of the intestine and thus enhancing the ability to absorb nutrients. Goblet cells, the major secretory cell in the superficial epithelium, produce and store large amounts of mucus and mucins, which functions for protecting intestine from mechanical damage (Cook et al., 2017). Results in the present study showed that fish fed diets with shrimp paste supplemented had more goblet cells, which suggested shrimp paste may have influence on protective effect of snakehead in intestine.

As for the antioxidant capacity, T-AOC is an overall indicator of the antioxidant status of an individual, on behalf of the level of non-enzyme and enzyme antioxidant in the organism (Xiao et al., 2004). As one of the important antioxidant enzymes, superoxide dismutase (SOD) is an important endogenous antioxidant for protection against oxidative stress and the first enzymes to respond against oxygen radicals (Winston and Di Giulio, 1991). Malondialdehyde (MDA) is a product of lipid peroxidation, through crosslinking with the nucleophilic groups of nucleic acids, amino phospholipids and proteins (Buege and Aust, 1978). The results showed that the MDA between the two diets with or without shrimp paste supplemented were not significantly different when no stress appeared in the present. The present results indicated that snakehead did not suffer from oxidative stress because of the dietary inclusion with shrimp paste.

Reports showed that there are biologically active peptides in protein hydrolysate with immuno-stimulating properties which is produced during the processing procedure (Daoud et al., 2005; Kotzamanis et al., 2007). NF-κB is a pleiotropic transcription factor, which is involved in diverse physiological and pathological processes including infection, inflammation and immunity (Karin and Greten, 2005). NF-κB consists of hetero- and homo-dimeric complexes of members in the Rel family of proteins, composed of p50, p52, p65 (RelA), c-Rel, and RelB (Liu et al., 2008). Activation of NF-κB signal pathway involved in the isolation of the inhibitor of κB (IκB) and led to the NF-κB complex translocating into the nucleus, thus promoted the expression level of NF-κB responsive genes, including IL-6, IL-1β and TNF-α (Moore et al., 1993). As a member of the family of chemokine, IL-8 has the chemotactic properties of leukocyte and lymphocyte and exert an effect on the initiation and amplification of acute inflammatory reaction in the chronic inflammatory process as a pre-inflammatory cytokine (Heinzmann et al., 2004; Hu et al., 2016). As a nuclear hormone receptor, GR is a member of the superfamily of ligand-activated transcription factors (Ning et al., 2019) and can cross talk with NF-κB pathways and thus make difference to the expression levels of inflammatory genes (Helen et al., 2004). The present study showed that fish fed diets with shrimp paste supplemented did not significantly up-regulate expression of GR, IκB, P65 and IL8 snakehead, indicating that shrimp paste exert no effect on immunity.

## Conclusion

In conclusion, dietary shrimp paste supplementation improved the growth performance of snakehead by enhancing FI, while made no difference to antioxidant capacity and non-specific immunity.

## Data Availability

All datasets generated for this study are included in the manuscript and/or the supplementary files.

## Ethics Statement

All experimental procedures were conducted in conformity with institutional guidelines for the care and use of laboratory animals in Sun Yat-sen University, Guangzhou, China, and conformed to the National Institutes of Health Guide for Care and Use of Laboratory Animals (Publication No. 85-23, revised 1985).

## Author Contributions

JN, YL, and LT designed the study. JX, HF, and SL carried out the rearing work. JX and JN analyzed the results. JX wrote the manuscript with contributions from all other authors.

## Conflict of Interest Statement

The authors declare that the research was conducted in the absence of any commercial or financial relationships that could be construed as a potential conflict of interest.
